# Wearable Monitoring and Interpretable Machine Learning Can Objectively Track Progression in Patients during Cardiac Rehabilitation

**DOI:** 10.3390/s20123601

**Published:** 2020-06-26

**Authors:** Hélène De Cannière, Federico Corradi, Christophe J. P. Smeets, Melanie Schoutteten, Carolina Varon, Chris Van Hoof, Sabine Van Huffel, Willemijn Groenendaal, Pieter Vandervoort

**Affiliations:** 1 Mobile Health Unit, Faculty of Medicine and Life Sciences, Hasselt University, 3500 Hasselt, Belgium; Christophe.smeets@uhasselt.be (C.J.P.S.); Melanie.schoutteten@uhasselt.be (M.S.); pieter.vandervoort@zol.be (P.V.); 2 Future Health Department, Ziekenhuis Oost-Limburg, 3600 Genk, Belgium; 3 imec the Netherlands/Holst Centre, 5656AE Eindhoven, The Netherlands; Federico.corradi@imec.nl (F.C.); Willemijn.groenendaal@imec.nl (W.G.); 4 KU Leuven, Department of Electrical Engineering (ESAT), STADIUS Center for Dynamical Systems, Signal Processing and Data Analytics, 3001 Leuven, Belgium; carolina.varon@esat.kuleuven.be (C.V.); chris.vanhoof@imec.be (C.V.H.); sabine.vanhuffel@esat.kuleuven.be (S.V.H.); 5 TU Delft, Department of Microelectronics, Circuits and Systems (CAS), 2600AA Delft, The Netherlands; 6 imec vzw Belgium, 3001 Leuven, Belgium; 7 Department of Cardiology, Ziekenhuis Oost-Limburg, 3600 Genk, Belgium

**Keywords:** wearable sensor, machine learning, physical fitness assessment, cardiac rehabilitation, patient progression monitoring

## Abstract

Cardiovascular diseases (CVD) are often characterized by their multifactorial complexity. This makes remote monitoring and ambulatory cardiac rehabilitation (CR) therapy challenging. Current wearable multimodal devices enable remote monitoring. Machine learning (ML) and artificial intelligence (AI) can help in tackling multifaceted datasets. However, for clinical acceptance, easy interpretability of the AI models is crucial. The goal of the present study was to investigate whether a multi-parameter sensor could be used during a standardized activity test to interpret functional capacity in the longitudinal follow-up of CR patients. A total of 129 patients were followed for 3 months during CR using 6-min walking tests (6MWT) equipped with a wearable ECG and accelerometer device. Functional capacity was assessed based on 6MWT distance (6MWD). Linear and nonlinear interpretable models were explored to predict 6MWD. The t-distributed stochastic neighboring embedding (t-SNE) technique was exploited to embed and visualize high dimensional data. The performance of support vector machine (SVM) models, combining different features and using different kernel types, to predict functional capacity was evaluated. The SVM model, using chronotropic response and effort as input features, showed a mean absolute error of 42.8 m (±36.8 m). The 3D-maps derived using the t-SNE technique visualized the relationship between sensor-derived biomarkers and functional capacity, which enables tracking of the evolution of patients throughout the CR program. The current study showed that wearable monitoring combined with interpretable ML can objectively track clinical progression in a CR population. These results pave the road towards ambulatory CR.

## 1. Introduction

Cardiovascular diseases (CVD), a general term encompassing a collection of heart and blood vessels-related disorders, are the leading cause of mortality worldwide [1]. The increasing prevalence of CVD, caused by an aging and growing population, shows that there is a high need for prevention programs in order to improve long-term outcomes [2]. Cardiac rehabilitation (CR) is a highly recommended secondary prevention measure and is considered to be a key component in current treatment strategies [3,4,5]. Unfortunately, most patients eligible for CR will not participate or complete the program due to referral issues, enrolment problems, or suboptimal completion rates [6,7]. A possible solution to improve participation rates is to move to home-based CR, which relies on remote monitoring and coaching strategies [8]. Moreover, the same remote monitoring strategies can further augment preventive cardiology practices by monitoring the patients’ disease status at home, as early signs of worsening can be detected [9]. However, the complexity of CVD makes reliable remote monitoring challenging. Most CVD are complex multi-system clinical syndromes and there is no single quantitative parameter available that captures the complexity of the information required to assess disease status. Therefore, a multi-parametric approach is necessary to accurately reflect the physiological condition of CVD patients.

A proper interpretation of physical fitness is essential to accurately follow-up a patient’s progression throughout a CR program. Various wearable sensors that can track a variety of physiological signals, reflective of physical fitness, are available today and their implementation within a cardiac population has been investigated extensively. Previous research suggested that free-living step count, measured by commercially available activity trackers, could function as an objective measure for functional classification in heart failure patients [10,11]. Thijs I. et al. showed that wearable technology, in the form of wrist worn devices, could function as an assessment tool of physical activity at home during rehabilitation after cardiac surgery [12]. However, the problem that arises in free-living conditions is the unsupervised assessment of physical activity, making proper interpretation challenging. Other research groups developed wearable systems to monitor exercise capacity during a submaximal exercise test. The submaximal 6-min walking test (6MWT) is easy to perform and reflects the ability to perform ordinary daily activities. Several studies focused on the added value of accelerometer-derived parameters in assessing performance during a 6MWT [13,14,15]. The results of these studies can contribute to the translation of a submaximal exercise test to assess functional capacity in patients towards an in-home environment. Moreover, adding extra features, e.g., heart rate (HR) estimation by means of photoplethysmograph (PPG) or electrocardiogram (ECG), in the follow-up of patients’ health status is continuously being examined [16,17]. The advantage of monitoring multiple features simultaneously is that it improves the accuracy of outcome measures [18].

Wearable sensor technology enables the ability to collect huge amounts of data, which can be used to optimize healthcare strategies to the patients’ needs and the changing socioeconomic system. The challenge to correctly interpret this large volume of data in a clinically meaningful and reliable manner remains [19]. Machine learning (ML) methods are increasingly being used in healthcare to tackle the complexity of diseases, patients, multi-parameter signals, and to take on the big data challenges [20]. Many possibilities of ML exist in the field of cardiovascular medicine to further optimize and personalize care [21]. Moreover, it allows to use a large collection of variables in a hypothesis free-approach to enable data-driven discovery [20]. Again, a standardized approach in a controlled environment is necessary to enable correct interpretation linked to the disease status of patients, thereby lowering the chances of false alerts in future use and preventing needless worrying by patients.

The goal of the present study was to investigate whether a multi-parameter sensor could be used during a standardized activity test in a controlled environment to interpret functional capacity in the longitudinal follow-up of CR patients. Moreover, it is investigated whether the combination of physiologically relevant features could function as a surrogate for functional capacity within a CR population.

## 2. Materials and Methods

### 2.1. Study Population

A total of 129 cardiovascular patients, following a multidisciplinary CR program at a single tertiary care center (Ziekenhuis Oost-Limburg, Genk, Belgium) were recruited for the study. Patients with heart failure and reduced ejection fraction, heart failure, and preserved ejection fraction and cardiovascular patients with a left ventricular ejection fraction less than or equal to 55% were eligible to participate. In addition, eligible patients were at least 18 years of age. The main exclusion criteria were inability to exercise due to orthopedic or neurological limitations. The group of patients who participated in this study were representative for the typical CR population. The study complied with the Declaration of Helsinki and the local ethical committee approved the study protocol (reference number: B371201423023). All subjects gave written informed consent prior to participation in the study.

### 2.2. Multidisciplinary CR Program

After a cardiovascular-related hospitalization, patients were referred to the multidisciplinary CR program. Patients had to follow a total of 45 ambulatory rehabilitation sessions, consisting of both aerobic and resistive exercises, at a frequency of three 1-h sessions a week. Moreover, dietary sessions, psychological support, and social consultations were offered throughout the multidisciplinary program. Functional capacity was assessed by a cardiopulmonary exercise test (CPET) at baseline and at end-of-study. The HR achieved at 90% of ventilator threshold during CPET was chosen as target HR during aerobic training. Resistive training was performed at 50–80% of one repetition maximum. Training intensity was increased every two weeks based on patient performance.

### 2.3. Study Design

At study entry the baseline characteristics, medical history, clinical data, treatment scheme, echocardiography data, and CPET results were collected from the electronic medical record. Every patient performed a 6MWT at baseline (start of the rehabilitation program). Next, a follow-up 6MWT was performed every three weeks, for four times in total. This resulted in five 6MWTs per patient collected throughout the study protocol. The 6MWT was performed according to a standardized protocol [22]. Prior to the 6MWT, patients were at rest for 5 min to achieve a resting HR. Additionally, a recuperation phase of 5 min was included after the 6MWT to achieve recovery HR. The distance walked after 6 min (6MWD) was recorded and was used to assess functional activity throughout the CR program. During the 6MWT, patients were equipped with a wearable multi-parameter device. The device was attached to the chest at the level of the lower sternum. The reliability and usability of the device was already proven in a previous study [23]. The wearable device was equipped with the Multi Sensor Integrated Circuit chip (MUSEIC, imec the Netherlands, Eindhoven, The Netherlands), supporting a wide range of sensor modalities, including ECG (Fs = 512 Hz) and accelerometer data (Fs = 32 Hz). The two electrodes were positioned according to lead II of Einthoven’s triangle.

### 2.4. Feature Extraction

The ECG and accelerometer data retrieved from the wearable device were divided into three different parts for each measurement. A 5-min resting phase (prior to walking), a 6-min walking phase, and a 5-min recuperation phase (after walking). Features are calculated independently for ECG and for accelerometer data. Features for each of the three different phases of the 6MWT were obtained. Therefore, no downsampling or upscaling of ECG or accelerometer data was performed. At first, artefacts within the ECG signal were automatically detected and removed after visual inspection [24]. Next, an initial automatic R-peak detection was performed using an algorithm described in [25] and wrong detections were manually corrected. The R-peaks were used to generate the HR, from which the HR parameters were calculated. All the parameters were smoothed using a non-overlapping sliding window of 150 ms in which the local HR was calculated. The 150 ms sliding window was chosen to match the width of the widest possible QRS complex. Additionally, by calculating a local average HR, the effects of ectopic beats on the subsequent computation of HR parameters is mitigated. Next, the local average HR values were used to deduce HR parameters that comprise the information of specific periods into a single output parameter. Resting HR (HR_rest_) was calculated by averaging the HR values during the 5-min lasting resting phase. The maximal HR (HR_max_) was extracted during the walking phase, by finding the maximum value of the local HR averages in the final 2 min of the recording. The average HR (HR_avg_) is also obtained by averaging the HR over the local average values of the 16 min recording. The chronotropic response was equal to the difference between HR_max_ and HR_rest_ and represented the ability of the heart to respond to exercise. Time to recuperation was calculated starting 5 s after walking, as the time required for the HR to decrease 66.6% of the range from HR_max_ to HR_rest_. The accelerometer data was used to estimate the patient’s effort during the walking phase of the 6MWT. Effort has previously been used as a measure of physical activity intensity [26,27,28]. Three non-overlapping time windows of 2 min length each, as well as for the full 6 min test duration were used to calculate the effort. The following formula was used to estimate the effort:

(1) $$\mathrm{effort}\;=\sqrt{\overset{n}{\underset{k=0}{\sum }}\left(X_{k}{}^{2}+Y_{k}{}^{2}+Z_{k}{}^{2}\right)}$$

The total number of accelerometer sample points considered is represented by *n* (*n* = Fs * recording time), $X_{k}$ is a vector representing the acceleration along X axis, while the other axes are represented, respectively, by $Y_{k}$, and $Z_{k}$ vectors. The anthropometrics features were retrieved from the patients’ electronic health records.

### 2.5. Linear Regression Model 

To determine the strongest predictors of 6MWD a stepwise linear regression model was built (Statistics and Machine Learning Toolbox, MATLAB, Version R2018a, Mathworks, Natick, MA, USA). Performance and goodness of fit of the model were assessed using the root-mean-square error (RMSE) and R-squared measures. The predictors that were included into the model were anthropometric-, ECG-derived, and accelerometer-derived features. The anthropometrics features were age, height, weight, and gender. Features derived from the electrocardiogram signal were HR_max_, HR_rest_, HR_avg_, chronotropic response, time to recuperation, and time to HR_max_ starting from the beginning of the 6MWT. Effort, an accelerometer-derived feature, together with HR parameters can function as an objective measure of functional capacity.

### 2.6. Machine Learning Model Derivation

Nonlinear relations are usually difficult to be captured by ANOVA and other linear methods. Therefore, a nonlinear model for predicting the 6MWD after a single 6MWT was explored with the selected sensor-derived features. The dataset was divided in two sets; 80% of the subjects were used to train our model while the remaining 20% of the patients were used for model validation. A 20-fold-validation was performed to measure prediction error. The performance of different support vector machine (SVM) regression models with functional capacity as target output were compared. Different kernel types were used, i.e., radial basis function (RBF), linear, polynomial of the 2nd order, 3rd order, and 4th order. The RBF kernel is also known as the universal approximator. In other words, it is a kernel that can approximate any other kernel depending on the sigma parameter. Sigma was optimized. In addition, the comparison of the kernels was extended to six feature combinations. The features used include height, chronotropic response, and effort (fitrsvm, Statistics and Machine Learning Toolbox, MATLAB, Version R2018a, Mathworks, Natick, MA, USA). The SVM regression model uses radial basis function kernels, with automatic scaling and enables to work with and predict continuous output variables. The kernel scaling divides all elements of the predictor matrix by the value of the kernel scale. Next, the appropriate kernel to compute the Gram matrix was applied. The kernel scaling was obtained using a heuristic procedure, which exploits subsampling, and the results can slightly vary at each iteration. However, no significant differences were noted in the 20 times that the algorithms ran. The model used was the Iterative Single Data Analysis as presented by Kecman et al. [29].

### 2.7. Feature Analysis and Tracking

Next, a state-of-the-art embedding technique that allows to visualize high dimensional data into a simple chart was used. To this end, the t-distributed stochastic neighboring embedding (t-SNE) technique was exploited [30] (Statistics and Machine Learning Toolbox, MATLAB, Version R2018a, Mathworks, Natick, MA, USA). It is a technique used for dimensionality reduction enabling the identification of relevant patterns in large datasets by creating a low dimensional graph while preserving the structure of the high dimensional data. In addition, this algorithm works in an unsupervised fashion, this means that no label or any a-priori knowledge was used to compute the mapping. Chronotropic response, effort, and session number were used as input features to create a composite of these three dimensions for each individual 6MWT. Next, to study the relation of these composites with other anthropometric and ECG-derived features, the plots were color-coded.

## 3. Results

### 3.1. Functional Capacity

Of the 129 patients that were included in the study, 89 patients completed the total study protocol. Forty patients were excluded from analysis due to failure to complete the total study protocol because of health-related problems, lack of motivation, and work or family commitment. Patients showed an increase in functional capacity throughout the CR program based on the results of the consecutive 6MWTs. A significant mean increase of 101 ± 59 m (*p* < 0.001) in 6MWD between baseline and end-of-study was seen. Patient characteristics and functional capacity measures are shown in Table 1.

### 3.2. Linear Regression Model 

**Figure 1:**
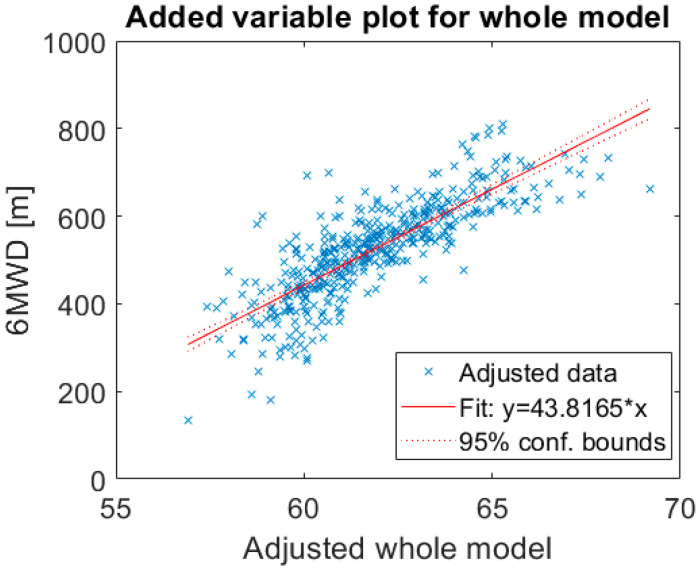
Added variable plot for the whole model. The adjusted whole model visualizes the fit of two independent variables (effort and chronotropic response) against the dependent variable. 6MWD, 6-min walking distance. R-squared: 0.661, *p*-value < 0.001.

Linear regression analysis was performed using stepwise variable selection procedures to identify the best predictors of exercise capacity measured during a standardized 6MWT. Anthropometric, ECG- and accelerometer-derived features were included as independent variables in the model with 6MWD as outcome measure. Non-predictors were removed to obtain a simpler model with fewer predictors while a similar predictive accuracy was maintained. Chronotropic response and effort were found to be the most significant predictors of 6MWD. The determination coefficient R^2^ of the model was 0.661, which indicates that the 6MWD can be reasonably determined by the chronotropic response and effort measured during the 6MWT. In other words, by using these two predictor variables 66% of variation in the 6MWD can be defined, the RMSE was 64.8 m. The overall model prediction is presented as an adjusted variable plot to visualize the fit of the independent variables, chronotropic response and effort, versus the dependent variable 6MWD (Figure 1).

### 3.3. Machine Learning Model Derivation

Chronotropic response and effort were the most relevant features selected by the stepwise linear regression analysis. Next, the performance of different SVM models was investigated to further analyze the relationship between feature combinations and the 6MWD. Table 2 shows the MEA ± SD on the test samples for predictions with different kernel types in SVM. RBF kernels perform better than other kernel types for most feature combinations. Additionally, based on the RBF kernel outcome, it becomes clear that adding chronotropic response and height to IMU effort, improves the performance of the SVM, meaning that both features have additional value. Based on MEA ± SD alone, the model combining the three features performs best. However, the difference between the chronotropic response + IMU effort model (42.8 m ± 36.8 m) and the chronotropic response + IMU effort + height model (37.7 m ± 36.2 m) is small. Moreover, height should be considered as a confounding factor as it remains stable throughout the different measurement sessions.

**Figure 2:**
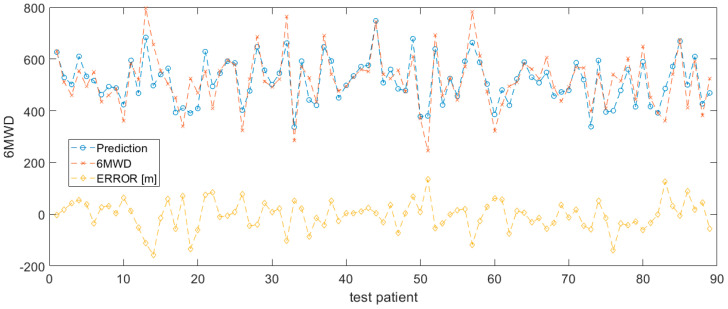
6MWD prediction based on accelerometer derived effort and chronotropic response.

The model performance was evaluated with random sampling by running 20-fold-validation. The patient data was split into a training and validation set with a splitting percentage of 80% train—20% validation. No significant difference was observed. Figure 2 shows the results of the 6MWD prediction for 20% of the patients in the validation set for a single random split (*n* = 18 patients, accounting for 89 6MWTs in total). The prediction curve (blue) approaches the trend of the actual 6MWD data (red), indicating a good performance of the SVM predictor, using only chronotropic response and accelerometer-derived effort as input features. With the exception of some 6MWT measurement sessions, the error on the 6MWD prediction is kept within reasonable ranges (yellow).

**Figure 3:**
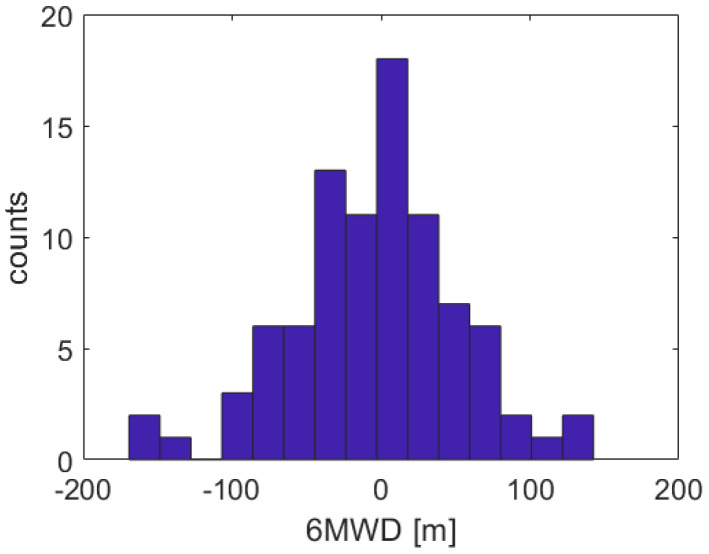
Error distribution in 6MWD prediction. Mean average error is equal to 42.5 m (±35.5 m).

Figure 3 shows the distribution of the error for the prediction of the 6MWD performed by the SVM model on the validation set. The mean average error was 42.5 m (±35.5 m).

### 3.4. Feature Analysis and Tracking

A nonlinear dimensionality reduction technique, t-SNE, incorporates all relevant features for each individual patient measurement session while plotting sessions with more similar features closer to each other. In addition, it facilitates the visualization of relevant patterns within a high dimensional dataset. Chronotropic response and accelerometer-derived effort were used as input features, together with 6MWT session number, representative for the moment in rehabilitation. The axes do not strictly represent distance or time but are the results of a composite projection of features in a 3D space. Each point in the 3D graphs obtained in the t-SNE represent a composite of dimensions for each individual 6MWT per patient. To visualize and study the relation of variables, not included as input features, the composite points were color-coded. A color-coded plot for anthropometric, ECG- and accelerometer-derived features was created. However, due to the high dimensional character of the available dataset, only a selection of color-coded plots, representative for the most relevant features, is shown.

**Figure 4:**
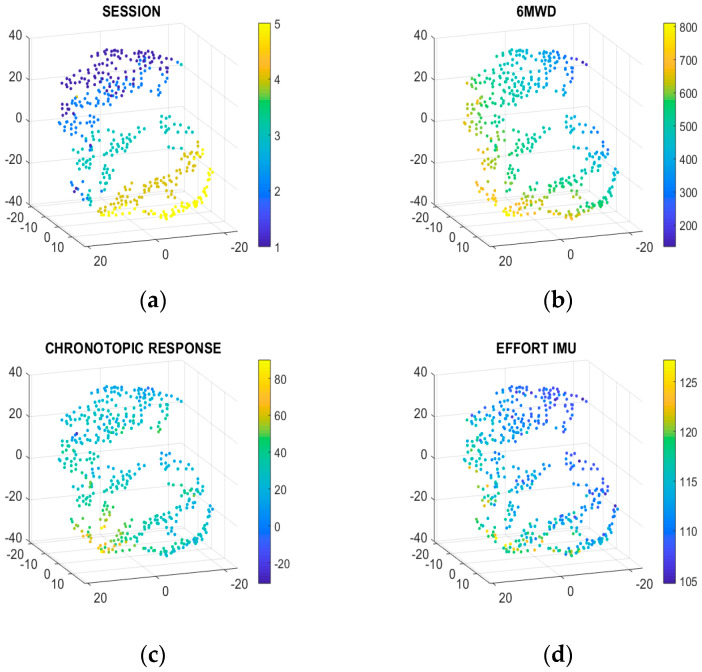
(**a**) t-distributed stochastic neighboring embedding (t-SNE) projection, colors represent rehabilitation time (session number) (upper left graph); (**b**) t-SNE projection, colors represent distance. Note that distance has not been used as input feature in the model, but it has only been used to color the map (upper right graph); (**c**) t-SNE projection, colors represent chronotropic response (bottom left graph); (**d**) t-SNE projection, colors represent effort during the last 2 min of the 6MWT (bottom right graph). The axes do not represent distance or time but are the results of a composite projection of features in a 3D space.

Figure 4 shows the selection of color-coded plots visualizing the relation of session number, distance, chronotropic response, and effort with the composite points. Figure 4a shows points color-coded by measurement session number to be able to investigate the relation between the chronotropic response, effort, and the actual moment in time in the rehabilitation program. This graph clearly represents the role of session number as an input feature. All patients performed five 6MWTs throughout CR, visualized by five colors ranging from dark blue to yellow. The composite points representative for baseline 6MWT are clustered at the top of the graph (blue points), while the composite points for the end-of-study 6MWT session are clustered at the bottom right part of the graph (yellow points). The position of each composite point is preserved in the other graphs, however the color-code is different for every feature that was studied. Figure 4c,d visualizes the relationship between, respectively, the chronotropic response and effort and the composite points. The yellow color stands for high chronotropic response and high effort, both indicators of good exercise capacity. It appears that higher chronotropic responses and high effort reside in a subspace which is close to the high performance 6MWD. The 6MWTs characterized by a high effort and high chronotropic are clustered at the bottom left side (yellow points) as shown in Figure 4c,d. When the same composite points are studied in the graph that is color-coded for 6MWD, it appears that these points are characterized by a high 6MWD (yellow) (Figure 4b). Whereas, sessions performed with low effort and low chronotropic response are located at the right side of the graph (blue points). These composite points are characterized by low 6MWD as seen in Figure 4b.

It is important to note that in Figure 4b 6MWD was only used to color-code the composite points, 6MWD was not used as an input feature to create the 3D graph.

### 3.5. Rehabilitation Tracking

**Figure 5:**
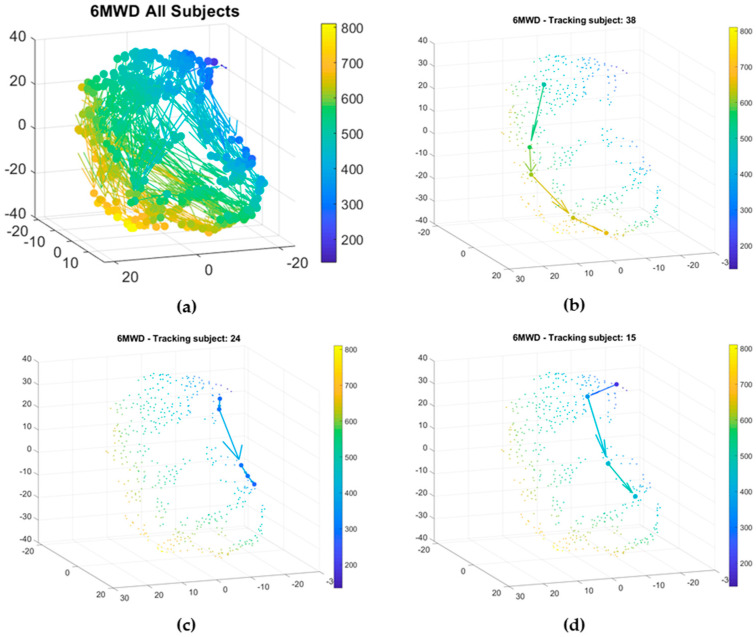
(**a**) Tracking all patients during the rehabilitation program (top left); (**b**) tracking of rehabilitation for patient 38. The 6MWD has increased from about 600 m (green) to more than 700 m (yellow) between the first and last session of the rehabilitation program (top right); (**c**) tracking of patient number 24. This patient shows a small difference in the 6MWD between the start and the end of the rehabilitation program (bottom left); (**d**) tracking of patient number 15. This patient shows an increase in 6MWD between baseline and the second 6MWT, stable afterwards (bottom right).

Figure 5a shows the rehabilitation trajectories for all subjects, color-coded by the 6MWD. The patients improve in functional capacity throughout the CR program, as visible by the increase in 6MWD and thus change in color. All patients will move from the upper to the lower part in space. Subjects who show a large improvement throughout CR are characterized by a change in color-coding (blue to yellow). Each patient is characterized by a unique trajectory. Figure 5b shows the trajectory of a patient who increases more than 100 m in 6MWD throughout CR. According to the subgroups present in Figure 4b,c, this patient can be characterized by an increase in chronotropic response and effort throughout CR, suggesting that the patient’s heart responds better to exercise at the end of CR compared to the start. Patient number 24 shows a small increase in 6MWD throughout CR (Figure 5c). The patient’s chronotropic response and effort remain low. The lack in improvement can be caused by the inability of the heart to adapt to exercise, as seen in the low chronotropic response values. Figure 5d shows the trajectory of patient number 15 throughout the CR program. Initially, an increase in 6MWD is seen between baseline and the second 6MWT, but the improvement stabilizes during the following measurements. Again, this patient is characterized by a low chronotropic response and effort value throughout CR. For these specific patients, a lower response to CR, represented by a low increase in distance walked throughout the program, is characterized by a lower chronotropic response and lower effort.

## 4. Discussion

### 4.1. Main Findings

The multifactorial complexity of CVD makes remote monitoring of patients and the translation of CR to an in-home setting challenging. Multi-parameter devices combined with interpretable AI can help in tackling these problems. The current study therefore investigated the usability of a multi-parameter sensor in a CR population. Moreover, the combination of physiologically relevant features as a surrogate for functional capacity was studied. The main findings of the current study are (1) the combination of chronotropic response and effort, both sensor-derived biomarkers, can function as a surrogate for functional capacity, captured during a standardized 6MWT in a CR population. (2) A 3D representation of these combined features enables the interpretation of functional capacity and together with the SVM regression model output, pave the road towards the implementation of explainable machine learning techniques by healthcare personnel. (3) In addition, these visualization techniques enable the follow-up of progression throughout CR, thereby opening up the possibility to move rehabilitation towards an in-home setting.

### 4.2. Linear and Nonlinear Regression Models

The 6MWT is a submaximal exercise test that is often used in cardiopulmonary patients to evaluate physical functional capacity. The outcome measure, the 6MWD, is representative for the ability of these patients to perform daily activities. Accelerometers have been proposed to measure physical performance during the 6MWT. Jehn et al. showed that both step frequency and activity counts, representative for effort, were strongly correlated with 6MWD [15]. In a subsequent study Jehn et al. showed that step count and walking speed, measured during 6MWTs in their home surroundings, were significantly correlated with 6MWD in controlled environment [14]. Other studies focused on investigating the added value of multiparametric monitoring. Lin et al. proposed a system to monitor cardiopulmonary parameters, i.e., HR and breathing rate, while obtaining precise walking information, i.e., walking speed and acceleration, during a 6MWT [13]. A difference in these parameters was seen between a smoker and nonsmoker group, indicating a difference in cardiopulmonary performance. Altini et al. went even further and estimated functional capacity in free-living conditions in a healthy population by combining HR and accelerometer-derived parameters measured by a wearable device [31]. By including these predictors into the model, they were able to explain 76% of the variance in estimated functional capacity. The regression model in our study, with chronotropic response and effort as predictors, explained 66% of the variance in estimated 6MWD, representative for functional capacity. Both studies presented models that gave a reasonably good prediction of functional capacity based on solely using accelerometer- and ECG-derived features as input. The difference in model performance can be explained by the difference in study population. A CR population is characterized by a large diversity among patients, with physical fitness levels ranging from a very low functional capacity to a functional capacity approaching the fitness levels of healthy subjects. As the CR patient population is often diverse and complex, the nonlinear relationship between the aforementioned features was further investigated. Therefore, a SVM regression model was trained to study the nonlinear relationships between the features selected by the regression analysis and the 6MWD. A model including effort, chronotropic response, and height showed best performance. The performance only slightly decreased when omitting height. Therefore, the model only containing features derived from the wearable sensor data, were compared to existing literature. Juen et al. and Salvi et al. both used machine learning techniques to predict 6MWD based on solely accelerometer-derived features in a pulmonary patient population [32,33]. Juen et al. was able to predict the distance with an error rate of 3.78%, while Salvi et al. made a slight underestimation of 2.01 m (±7.84 m). These models show a better prediction of 6MWD when compared to our overestimation of 42.8 m (±36.8 m). However, as mentioned previously the CR population is characterized by a diversity in patients which is also represented in the large range of walking distances among patients. Despite this large variety in distances, the difference between the distance estimated by the SVM model and the actual distance was for most patients close to 50 m, which is considered to be the clinically significant threshold for detecting changes in disease status [34]. Moreover, chronotropic response as an extra input feature contributed to the ability to predict functional capacity in the CR population. This is in line with previous studies, which have already shown that chronotropic response is a significant predictor of training response during a CR program [35,36]. If CR is moved to an in-home setting, proper context interpretation is necessary. It is difficult to correctly interpret the progression, performance, and motivation of the patients based on 6MWD alone. Therefore, adding ECG features contributes to a proper interpretation of functional capacity. In addition, the information of the response of the heart to exercise and the intensity wherewith the exercise was performed, contributed to the remote optimization of the training schedule of the patient. Future research can focus on the role of the additional information extracted from the ECG signal when implemented in a remote rehabilitation setting.

### 4.3. 3D Visualization of Functional Capacity on Population and Personal Level

The 3D dimensional representation enables the visualization of the relations between chronotropic response, effort, and 6MWD. Even more, the contribution of chronotropic response and effort in the prediction of 6MWD is explained in these 3D models. On population level, a distinction between 6MWT performances can be made by only using these sensor-derived features. The high performance 6MWTs, i.e., long predicted distance, are characterized by high chronotropic response and high effort, while for low performance 6MWTs the opposite is true. The 3D visualization enables the personalized tracking of progression in functional capacity throughout CR. It enables the physician to not only track the patients’ progress, but it also allows to determine the contribution of chronotropic response and effort in the assessment of functional capacity. Moreover, it enables to compare the performance of different patients to one another. Therefore, being able to assess functional capacity paves the road towards home-based CR. This type of explainable and interpretable use of machine learning techniques are highly needed in healthcare settings as it enables to process large amounts of data but at the same time lets the physician comprehend the outcome. AI techniques are finding their way into the world of healthcare [21]. Machine learning approaches have been used to classify heart failure patients based on their disease status. In addition, in the field of imaging, machine learning techniques are being used more frequently [37,38,39]. The implementation of wearable technologies in CR are being investigated by validating the ability to correctly measure specific physiological features, such as heart rate [40,41]. Our results show the potential of machine learning and sensor technology to tackle the complexity of cardiovascular diseases, thereby facilitating, improving, and personalizing patient follow-up.

### 4.4. Limitations and Future Perspectives

Future studies should focus on measuring additional physiological relevant features, i.e., SpO_2_, respiration rate etc., and on studying their role in assessing functional capacity in order to further refine the models and to improve the prediction capacity of the models. Investigating whether the model prediction would improve when applied in specific subpopulations within the CR population, would also be of interest. However, this requires the inclusion of additional patients. In addition, although the 3D visualization enables the interpretation of the contribution of chronotropic response and effort in the assessment of functional capacity, it should be noted that a limitation to this technique is that the 3D visualization is not straightforward. Therefore, in the future an alternative to the 3D graphs should be considered for interpretation by the physicians.

## 5. Conclusions

To conclude, the main findings of the study were as follows: (1) the combination of sensor-derived biomarkers, i.e., chronotropic response and effort, can function as a surrogate for functional capacity in a CR population during a standardized 6MWT. (2) A 3D representation of these combined features facilitates the interpretation of this functional capacity surrogate. (3) These visualization techniques enable the follow-up of progression throughout a CR program by tracking the movement on patient level throughout the graph with respect to chronotropic response and effort. Finally, these findings pave the road towards the implementation of machine learning techniques that are interpretable by healthcare personnel and can facilitate the translation of CR to an in-home setting.

## Figures and Tables

**Table 1:** Patient characteristics and functional capacity assessed based on 6-min walking distance (6MWD).

Variable	Total Population (*n* = 89)
**Anthropometric Features**	
Male	65 (73%)
Age, yrs	63 ± 1
Height, m	1.72 [1.70–1.74]
Weight, kg	79.2 ± 1.4
BMI, kg/m^2^	26.7 ± 0.4
LV ejection fraction, %	46 [43–49]
**Comorbidities**	
Atrial fibrillation	22 (25%)
Hypertension	38 (43%)
Dyslipidemia	39 (44%)
Diabetes	12 (14%)
**NYHA Class**	
Class I	26 (29%)
Class II	44 (49%)
Class III	19 (21%)
**6MWD, m**	
Baseline	484 ± 96
1st follow-up	533 ± 100
2nd follow-up	564 ± 100
3rd follow-up	570 ± 103
End of study	585 ± 104
Baseline VO_2_ max, mL/kg/min	17.0 ± 5.1

BSA, Body Surface Area; LV, left ventricular; NYHA, New York Heart Association; 6MWD, 6-min walking distance.

**Table 2:** Kernel type comparison in support vector machine model.

Kernel Type	MAE ± STD	Features
RBF	42.8 m ± 36.8 m	IMU effort, chronotropic response
Linear	55.2 m ± 51.3	
Polynomial order 2	45.3 m ± 43.3 m	
Polynomial order 3	58.3 m ± 55 m	
Polynomial order 4	259.4 m ± 68 m	
RBF	40.1 m ± 39.1 m	IMU effort, height
Linear	67.6 m ± 62.5 m	
Polynomial order 2	257.3 m ± 70.3 m	
Polynomial order 3	98.6 m ± 77.5 m	
Polynomial order 4	284.1 m ± 92.6 m	
RBF	47.2 m ± 47.6 m	IMU effort
Linear	67.7 m ± 63.2 m	
Polynomial order 2	90.7 m ± 60.7 m	
Polynomial order 3	267.6 m ± 57.6 m	
Polynomial order 4	285.6 m ± 67.6 m	
RBF	37.7 m ± 36.2 m	IMU effort, chronotropic response, height
Linear	55.0 m ± 51.8 m	
Polynomial order 2	42.6 m ± 40.4 m	
Polynomial order 3	44.6 m ± 50.7 m	
Polynomial order 4	263.5 m ± 176 m	
RBF	60.0 m ± 58.7 m	Chronotropic response
Linear	131.0 m ± 110.4 m	
Polynomial order 2	75.7 m ± 71.4 m	
Polynomial order 3	83.2 m ± 71.7 m	
Polynomial order 4	89.9 m ± 81.1 m	
RBF	67.5 m ± 67.1 m	Chronotropic response, height
Linear	100.1 m ± 85.3m	
Polynomial order 2	65.5 m ± 59.3 m	
Polynomial order 3	193.9 m ± 123.1 m	
Polynomial order 4	250.5 m ± 408.4 m	

RBF, radial basis function; MAE, mean average error; SD, standard deviation.

## References

[1] Gregory A.R., Degu A., Kalkidan H.A., Solomon M.A., Cristiana A., Nooshin A., Hedayat A., Foad A.-A., Jemal A., Ahmed A. (2018). Global, regional, and national age-sex-specific mortality for 282 causes of death in 195 countries and territories, 1980–2017: A systematic analysis for the Global Burden of Disease Study 2017. Lancet.

[2] Frederix I., Dendale P., Schmid J.P. (2017). Who needs secondary prevention?. Eur. J. Prev. Cardiol..

[3] Ponikowski P., Voors A.A., Anker S.D., Bueno H., Cleland J.G.F., Coats A.J.S., Volkmar F., José R.G.-J., Veli-Pekka H., Ewa A.J. (2016). 2016 ESC Guidelines for the diagnosis and treatment of acute and chronic heart failure: The Task Force for the diagnosis and treatment of acute and chronic heart failure of the European Society of Cardiology (ESC)Developed with the special contribution of the Heart Failure Association (HFA) of the ESC. Eur. Heart J..

[4] Thomas R.J., Balady G., Banka G., Beckie T.M., Chiu J., Gokak S. (2018). 2018 ACC/AHA Clinical Performance and Quality Measures for Cardiac Rehabilitation: A Report of the American College of Cardiology/American Heart Association Task Force on Performance Measures. J. Am. Coll. Cardiol..

[5] Yancy C.W., Jessup M., Bozkurt B., Butler J., Casey D.E., Drazner M.H., Gregg C.F., Stephen A.G., Tamara H., James L.J. (2013). 2013 ACCF/AHA guideline for the management of heart failure: A report of the American College of Cardiology Foundation/American Heart Association Task Force on Practice Guidelines. J. Am. Coll. Cardiol..

[6] Dalal H.M., Doherty P., Taylor R.S. (2015). Cardiac rehabilitation. BMJ (Clin. Res. Ed.).

[7] Piepoli M.F., Corra U., Adamopoulos S., Benzer W., Bjarnason-Wehrens B., Cupples M. (2014). Secondary prevention in the clinical management of patients with cardiovascular diseases. Core components, standards and outcome measures for referral and delivery: A policy statement from the cardiac rehabilitation section of the European Association for Cardiovascular Prevention & Rehabilitation. Endorsed by the Committee for Practice Guidelines of the European Society of Cardiology. Eur. J. Prev. Cardiol..

[8] Thomas R.J., Beatty A.L., Beckie T.M., Brewer L.C., Brown T.M., Forman D.E., Franklin B.A., Keteyian S.J., Kitzman D.W., Regensteiner J.G. (2019). Home-Based Cardiac Rehabilitation. J. Cardiopulm. Rehabil. Prev..

[9] Vegesna A., Tran M., Angelaccio M., Arcona S. (2016). Remote Patient Monitoring via Non-Invasive Digital Technologies: A Systematic Review. Telemed. eHealth.

[10] Baril J.-F., Bromberg S., Moayedi Y., Taati B., Manlhiot C., Ross H., A Cafazzo J. (2019). Use of Free-Living Step Count Monitoring for Heart Failure Functional Classification: Validation Study. JMIR Cardio.

[11] Moayedi Y., Abdulmajeed R., Posada J.D., Foroutan F., Alba A.C., A Cafazzo J., Ross H. (2017). Assessing the Use of Wrist-Worn Devices in Patients With Heart Failure: Feasibility Study. JMIR Cardio.

[12] Thijs I., Fresiello L., Oosterlinck W., Sinnaeve P., Rega F., Goris J., Vogt F., Pietilä J. (2019). Assessment of Physical Activity by Wearable Technology During Rehabilitation After Cardiac Surgery: Explorative Prospective Monocentric Observational Cohort Study. JMIR mHealth uHealth.

[13] Lin B.-S., Jhang R.-J., Lin B.-S. (2019). Wearable Cardiopulmonary Function Evaluation System for Six-Minute Walking Test. Sensors.

[14] Jehn M., Prescher S., Koehler K., Von Haehling S., Winkler S., Deckwart O., Honold M., Sechtem U., Baumann G., Halle M. (2013). Tele-accelerometry as a novel technique for assessing functional status in patients with heart failure: Feasibility, reliability and patient safety. Int. J. Cardiol..

[15] Jehn M., Schmidt-Trucksäess A., Schuster T., Hanssen H., Weiß M., Halle M., Koehler F. (2009). Accelerometer-Based Quantification of 6-Minute Walk Test Performance in Patients with Chronic Heart Failure: Applicability in Telemedicine. J. Card. Fail..

[16] Henriksen A., Mikalsen M.H., Woldaregay A.Z., Muzny M., Hartvigsen G., A Hopstock L., Grimsgaard S., Sanders J., Wark P., Winfree K. (2018). Using Fitness Trackers and Smartwatches to Measure Physical Activity in Research: Analysis of Consumer Wrist-Worn Wearables. J. Med. Internet Res..

[17] Majumder S., Mondal T.K., Deen J. (2017). Wearable Sensors for Remote Health Monitoring. Sensors.

[18] O’Driscoll R., Turicchi J., Beaulieu K., Scott S., Matu J., Deighton K., Finlayson G., Stubbs J. (2018). How well do activity monitors estimate energy expenditure? A systematic review and meta-analysis of the validity of current technologies. Br. J. Sports Med..

[19] Wongvibulsin S., Martin S.S., Steinhubl S.R., Muse E.D. (2019). Connected Health Technology for Cardiovascular Disease Prevention and Management. Curr. Treat. Options Cardiovasc. Med..

[20] Shameer K., Johnson K.W., Glicksberg B.S., Dudley J.T., Sengupta T.B.A.P.P. (2018). Machine learning in cardiovascular medicine: Are we there yet?. Heart (Br. Card. Soc.).

[21] Gevaert A.B., Adams V., Bahls M., Bowen T.S., Cornelissen V., Dörr M., Hansen D., Mc Kemps H., Leeson P., Van Craenenbroeck E.M. (2019). Towards a personalised approach in exercise-based cardiovascular rehabilitation: How can translational research help? A ’call to action’ from the Section on Secondary Prevention and Cardiac Rehabilitation of the European Association of Preventive Cardiology. Eur. J. Prev. Cardiol..

[22] American Thoracic Society (2002). ATS statement: Guidelines for the six-minute walk test. Am. J. Respir. Crit. Care Med..

[23] Van Steenkiste T., Groenendaal W., Ruyssinck J., Dreesen P., Klerkx S., Smeets C., De Francisco R., Deschrijver D., Dhaene T. Systematic Comparison of Respiratory Signals for the Automated Detection of Sleep Apnea. Proceedings of the 2018 40th Annual International Conference of the IEEE Engineering in Medicine and Biology Society (EMBC).

[24] Varon C., Testelmans D., Buyse B., Suykens J.A.K., Van Huffel S. Robust artefact detection in long-term ECG recordings based on autocorrelation function similarity and percentile analysis. Proceedings of the 2012 Annual International Conference of the IEEE Engineering in Medicine and Biology Society.

[25] Moeyersons J., Amoni M., Van Huffel S., Willems R., Varon C. (2019). R-DECO: An open-source Matlab based graphical user interface for the detection and correction of R-peaks. bioRxiv.

[26] Bai J., Di C., Xiao L., Evenson K.R., Lacroix A.Z., Crainiceanu C.M., Buchner D.M. (2016). An Activity Index for Raw Accelerometry Data and Its Comparison with Other Activity Metrics. PLoS ONE.

[27] Colley R.C., Garriguet D., Janssen I., Craig C.L., Clarke J., Tremblay M.S. (2011). Physical activity of Canadian adults: Accelerometer results from the 2007 to 2009 Canadian Health Measures Survey. Health Rep..

[28] Migueles J.H., Cadenas-Sanchez C., Ekelund U., Nyström C.D., Mora-Gonzalez J., Löf M., Labayen I., Ruiz J.R., Ortega F.B. (2017). Accelerometer Data Collection and Processing Criteria to Assess Physical Activity and Other Outcomes: A Systematic Review and Practical Considerations. Sports Med..

[29] Kecman V., Huang T.-M., Vogt M. (2005). Iterative Single Data Algorithm for Training Kernel Machines from Huge Data Sets: Theory and Performance. Integration of Fuzzy Logic and Chaos Theory.

[30] Maaten L., Hinton G. (2008). Visualizing data using t-SNE. J. Mach. Learn. Res..

[31] Altini M., Casale P., Penders J., Amft O. (2016). Cardiorespiratory fitness estimation in free-living using wearable sensors. Artif. Intell. Med..

[32] Juen J., Cheng Q., Schatz B. (2015). A Natural Walking Monitor for Pulmonary Patients Using Mobile Phones. IEEE J. Biomed. Health Inform..

[33] Lynch J., Fang Q., Salvi D., Poffley E., Orchard E., Tarassenko L. (2020). The Mobile-Based 6-Minute Walk Test: Usability Study and Algorithm Development and Validation. JMIR mHealth uHealth.

[34] Rasekaba T., Lee A.L., Naughton M.T., Williams T.J., Holland A.E. (2009). The six-minute walk test: A useful metric for the cardiopulmonary patient. Intern. Med. J..

[35] Schmid J.-P., Zurek M., Saner H. (2012). Chronotropic incompetence predicts impaired response to exercise training in heart failure patients with sinus rhythm. Eur. J. Prev. Cardiol..

[36] Zweerink A., Van Der Lingen A.-L.C., Handoko M.L., Van Rossum A.C., Allaart C. (2018). Chronotropic Incompetence in Chronic Heart Failure. Circ. Heart Fail..

[37] Ahmad T., Lund L.H., Rao P., Ghosh R., Warier P., Vaccaro B., Dahlström U., O’Connor C.M., Felker G.M., Desai N.R. (2018). Machine Learning Methods Improve Prognostication, Identify Clinically Distinct Phenotypes, and Detect Heterogeneity in Response to Therapy in a Large Cohort of Heart Failure Patients. J. Am. Heart Assoc..

[38] Dey D., Slomka P.J., Leeson P., Comaniciu D., Shrestha S., Sengupta P.P. (2019). Faculty Opinions recommendation of Artificial Intelligence in Cardiovascular Imaging: JACC State-of-the-Art Review. Faculty Opin. Post Publ. Peer Rev. Biomed. Lit..

[39] Kao D.P., Lewsey J.D., Anand I., Massie B.M., Zile M.R., Carson P.E., McKelvie R.S., Komajda M., McMurray J.J., Lindenfeld J. (2015). Characterization of subgroups of heart failure patients with preserved ejection fraction with possible implications for prognosis and treatment response. Eur. J. Heart Fail..

[40] Alharbi M., Bauman A., Neubeck L., Gallagher R. (2016). Validation of Fitbit-Flex as a measure of free-living physical activity in a community-based phase III cardiac rehabilitation population. Eur. J. Prev. Cardiol..

[41] Etiwy M., Akhrass Z., Gillinov L., Alashi A., Wang R., Blackburn G., Gillinov S., Phelan D., Gillinov A.M., Houghtaling P.L. (2019). Accuracy of wearable heart rate monitors in cardiac rehabilitation. Cardiovasc. Diagn. Ther..

